# Exploring the perspectives of community members as research partners in rural and remote areas

**DOI:** 10.1186/s40900-020-0179-6

**Published:** 2020-01-30

**Authors:** Chelsea A. Pelletier, Anne Pousette, Kirsten Ward, Gloria Fox

**Affiliations:** 1 School of Health Sciences, University of Northern British Columbia, Prince George, Canada; 2 Promotion of Wellness in Northern BC (WINBC), Clinical Faculty, Northern Medical Program, University of British Columbia, Medical Staff, University Hospital of Northern BC, Northern Health, Prince George, Canada; 3 Present address: Department of Physical Therapy, University of British Columbia, Vancouver, Canada; 4 Population and Preventive Public Health, Northern Health, Prince George, Canada

**Keywords:** Qualitative methods, Qualitative health research, Patient-oriented research, Integrated knowledge translation, Patient and public involvement, Rural health, Population health

## Abstract

**Background:**

Community engagement in research has the potential to support the development of meaningful health promotion interventions to address health inequities. People living in rural and remote areas face increased barriers to participation in health research and may be unjustly excluded from participation. It is necessary to understand the process of patient and public engagement from the perspective of community members to support partnered research in underserved areas. The aim of this project was to increase understanding on how to include community members from rural and remote areas as partners on research teams.

**Methods:**

Using purposive sampling, we completed semi-structured interviews with a representative sample of 12 community members in rural and remote areas of northern British Columbia, Canada. Interviews were audio recorded and transcribed verbatim. Following an integrated knowledge translation approach, an inductive thematic analysis was completed to incorporate researcher and knowledge user perspectives.

**Results:**

The factors important to community members for becoming involved in research include: 1) relevance; 2) communication; and 3) empowering participation. The analysis suggests projects must be relevant to both communities and individuals. Most participants stated that they would not be interested in becoming partners on research projects that did not have a direct benefit or value for their communities. Participants expressed the need for clear expectations and clarification of preferred communication mechanisms. Communication must be regular, appropriate in length and content, and written in a language that is accessible. It is essential to ensure that community members are recognized as subject matter experts, to provide appropriate training on the research process, and to use research outcomes to support decision making.

**Conclusions:**

To engage research partners in rural and remote communities, research questions and outcomes should be co-produced with community members. In-person relationships can help establish trust and bidirectional communication mechanisms are prudent throughout the research process, including the appropriate sharing of research findings. Although this project did not include community members as research team members or in the co-production of this research article, we present guidelines for research teams interested in adding a patient or public perspective to their integrated knowledge translation teams.

## Plain English summary

To help make research more relevant to the public, it is important that researchers partner with community members. People living in rural and remote communities tend to be further away from where research typically takes place and may be excluded from participation as team members. The goal of this project was to understand how researchers can better work with or engage community members from rural and remote areas as partners on research projects. We talked to 12 community members with an interest in physical activity who live in northern British Columbia, Canada. Transcripts were analysed by researchers and knowledge users working in population health at the local health authority and a regional non-profit organization. We identified three factors that were important for research partnerships: relevance, communication, and empowering participation. Community members stated that they would not be interested in joining a research project that did not benefit their community. Participants also identified that they wanted to receive regular feedback about the research project, such as the findings, and to know that the results were used to create change. Community members should be recognized as the experts on the approach that would work best in their community, be offered training on the research process, and compensated appropriately. In rural and remote communities, it is especially important to focus on building trust and relationships in-person before beginning research partnerships as there is a history of researchers coming in from other areas (often urban centres), collecting data, and leaving.

## Background

With a growing focus on patient and public engagement in research, there is an increasing need to understand strategies and methods of partnering with community members on research teams. Funding bodies expect and frequently require engagement or demonstrated partnerships with patients and the public to be included in funding applications or to inform proposals. There are several national-level organizations that support and provide frameworks for integrated or engaged research approaches including the Strategy for Patient-Oriented Research (SPOR) from the Canadian Institutes for Health Research [1], the Patient-Centred Outcomes Research Institute (PCORI) in the United States [2], and National Institute for Health Research (NIHR) INVOLVE in the United Kingdom [3]. Although these national guidelines and frameworks provide principles to support patient and public engagement in research, they do not necessarily reflect the reality of particular communities or geographies. These organizations also have a predominant focus engaging with patient partners in a clinical environment and provide limited guidance for population health paradigms [4, 5].

Integrated knowledge translation is a research approach that advocates the co-production of research with knowledge user team members, typically defined as people or organizations that are likely to benefit from or who may use research findings in practice or to guide decision making [6]. Knowledge users may include, but are not limited to, research funders, health care providers, health system decision makers, advocacy organizations, patient groups, and/or members of the public [6, 7]. Although they represent an important knowledge user group, community members (or the public) are less commonly engaged on research teams, and the evidence base for community-centered population health research is fragmented [8–10]. Understanding concepts and strategies for community member engagement in health research locally will help research teams operationalize engagement in a way that supports and accounts for the characteristics, needs, and preferences of particular groups of people to develop best practice guidelines.

When compared to urban centres, rural and remote communities face increased challenges implementing effective health promotion interventions. Rates of noncommunicable disease tend to be higher in rural areas when compared to urban centres due to decreased participation in health-promoting behaviours, decreased access to health services, and social-ecological factors that may not be reflected in urban-centric programming [11, 12]. Patient and public engagement in research is proposed as a strategy to combat health inequities, but can only be successful if the proper groups are included and supported as full partners [13, 14]. Individuals living in rural communities can be considered ‘hard-to-reach’ as they are in settings where research does not typically take place [15]. For rural communities, there are specific barriers to research participation related to transportation, confidentiality, and culture [16]. To engage meaningfully with these groups, it is necessary to bring research to the communities where people live, dedicate time for personal relationship building, and establish trust between community members and researchers [15, 17]. The conceptualization of health differs between urban and rural communities [18], suggesting that researchers should not assume that what has worked in urban centres should be applied in engagement initiatives, research development, or health promotion interventions in rural communities.

### Setting the context: northern British Columbia (BC)

The provincial north of BC includes the northern two-thirds of the province, consists mainly of rural towns and small population centres, and is diverse in culture, geography, and access to health services. There are numerous health inequities when comparing the various health authorities in BC; the region served by Northern Health has a life expectancy 3.1 years lower than the provincial average and increased rates of noncommunicable disease [19, 20]. Similar to many other provinces in Canada, research institutions and universities are clustered in large urban centres of BC, which mostly lie close to the southern border. This creates distance between researchers, knowledge users, and communities. The University of Northern British Columbia (UNBC) is a research-intensive institution located in Prince George, BC, the largest population centre and unofficial capital of northern BC. Prince George is a northern and isolated medium sized population centre of approximately 78,675 people [21], on the traditional unceded territory of the Lheidli T’enneh peoples.

This project aims to understand how to facilitate the engagement of community members from rural and remote areas as patient or public partners on research teams. It is the intention that this work will identify strategies, mechanisms, and principles to support the development of a framework to engage community members on our integrated knowledge translation research team that focuses on physical activity in the context of northern, rural, and remote communities.

## Methods

The research team includes researchers (CP, KW) and knowledge users representing health system decision makers in population health (GF), a health care provider (AP), and the non-profit sector (AP). During project development, all team members completed the Foundations in Patient-Oriented Research course offered by the BC SUPPORT Unit [22]. This course introduces knowledge users, patients, and researchers to patient-oriented research, the research process, and strategies for working collaboratively on diverse research teams [23]. This training ensured that everyone on the team collaboratively learned the concepts essential to patient and public engagement in research and established a culture of growth among team members. All study protocols and materials, including the recruitment strategy, inclusion/exclusion criteria, recruitment matrix, interview schedule, research ethics application, data analysis, and knowledge translation outputs, were co-produced by researcher and knowledge user team members through regular in person meetings and circulated via email for approval until consensus was reached.

Twelve participants were recruited via email for semi-structured interviews using existing networks. Eligible participants included community members living in northern BC (defined as the Northern Health Region, see: https://www2.gov.bc.ca/gov/content?id=F220C3323A3B42D594A07A81947392BF) for a period of at least 1-year and with experience in their community related to physical activity and/or health research. Using purposive sampling, we aimed for a maximum variation sample [24, 25], seeking representation across a variety of lenses including community size, age, self-reported gender, geographical location (based on health service delivery area), ethnicity, experience with research, employment status, length of time living in northern BC, and education. Recruitment was guided using a recruitment matrix that plotted participants on a table based on community size and age. As participants were recruited and completed the interviews, we tracked representation back to our original targets and sought participants who met specific lenses or criteria as needed. Demographic information was collected with a questionnaire.

Interviews were conducted by a trained research assistant (KW) and at least one other author with experience in qualitative data collection. Interviews were conducted at a location of the participant’s choosing either in person or over the phone when travel was unfeasible. The majority of interviews occurred on the UNBC campus or in the participant’s workplace. Interviews were between 20 and 63 min in length (mean = 39 min); a combined total of 7 h and 54 min of data. All interviews were audio recorded and transcribed verbatim by the research assistant (KW). A pseudonym was assigned to each participant using a random name generator and any information that could potentially identify participants (e.g. community names, occupations, organization names) were removed. Anonymizing interview transcripts is particularly important for research with rural communities, due to the often dual and conflicting roles of community members [26]. All participants were given the opportunity to review and approved the final transcript of their interview.

Participants were invited to share their understanding of and any past positive or negative experiences with health research. The interview schedule also included questions and prompts to encourage participants to explain how researchers could best include community members as partners on research teams and what supports would best facilitate their engagement. Participants were also invited to share their experiences and understanding of physical activity; this analysis will be reported separately. Probing questions, such as providing examples of roles community members could play on research teams, were utilized to encourage detailed explanations.

Data were analyzed using an inductive thematic approach [27]. Coding was completed independently by three investigators (CP, AP, KW). The team met, discussed and refined the codes and patterns as they were identified in the data. Initial themes were identified and then discussed with the entire team for interpretation to include perspectives of both researchers and knowledge users. Final themes and subthemes were drafted based on the input from all team members and revised until consensus was reached. Field notes were used to provide context during data organization and theming. Identified themes, ideas, and experiences shared by participants were used to create a framework for community-partnered research to portray factors that should be considered by research teams interested in patient and public engagement. In addition, based on the learnings from both completing this integrated knowledge translation project and in our discussions with community members, our research team co-produced a set of principles and takeaway messages for both researchers and knowledge-users when partnering on research teams (presented as implications & future directions). A plain language summary was also co-produced by team members and shared with all participants and relevant stakeholders in our network (see Additional file 1).

All study procedures were approved by the UNBC a and Northern Health Research Ethics Committees. This project was funded by a Developing Northern Collaborations Award from the BC SUPPORT Unit, Northern Centre. All participants provided informed consent. For in-person interviews, written consent was obtained. In the case of telephone interviews, the participant information and consent form were sent via email prior to the scheduled interview and verbal consent was obtained.

## Results

The majority of participants (58%) were in the age category of 35–54 years and female (58%; Table 1). Half of participants (50%) reported completing post-graduate education. Six participants (50%) were currently working and six (50%) were retired but still actively engaged in their community through volunteer roles. Participants reported living in northern BC for anywhere from 5 to 52 years (average: 36.3 years), with many having grown up in the region. Six participants (50%) reported prior research experience, ranging from coordinating patient groups, assisting in participant recruitment, as a research team member, as a research participant, and as a component of their health professional degree program.

**Table 1:** Participant characteristics

Characteristic		Participants (*n* = 12)
Age (years)	< 35	-
	35–44	3 (25%)
	45–54	4 (33%)
	55–64	2 (17%)
	65–74	1 (8%)
	75 or older	2 (17%)
Gender	Male	5 (42%)
	Female	7 (58%)
Ethnicity	Caucasian	6 (50%)
	First Nations	2 (17%)
	Canadian	4 (33%)
Highest level of education	High School Only	1 (8%)
	Some post-secondary	5 (42%)
	Post-graduate	6 (50%)
Community size	< 1000	2 (17%)
	1000–5000	3 (25%)
	5000–10,000	1 (8%)
	10,000–20,000	-
	20,000–29,000	1 (8%)
	30,000–99,999	5 (42%)
Health service delivery area	Northern Interior	8 (67%)
	Northwest	2 (17%)
	Northeast	2 (17%)

Note: Population size based on BC Stats (2016). 2016 Census – population and housing – municipalities by regional district. Health Service Delivery Area in Northern Health Region (see: https://www2.gov.bc.ca/gov/content?id=F220C3323A3B42D594A07A81947392BF)

**Fig. 1:**
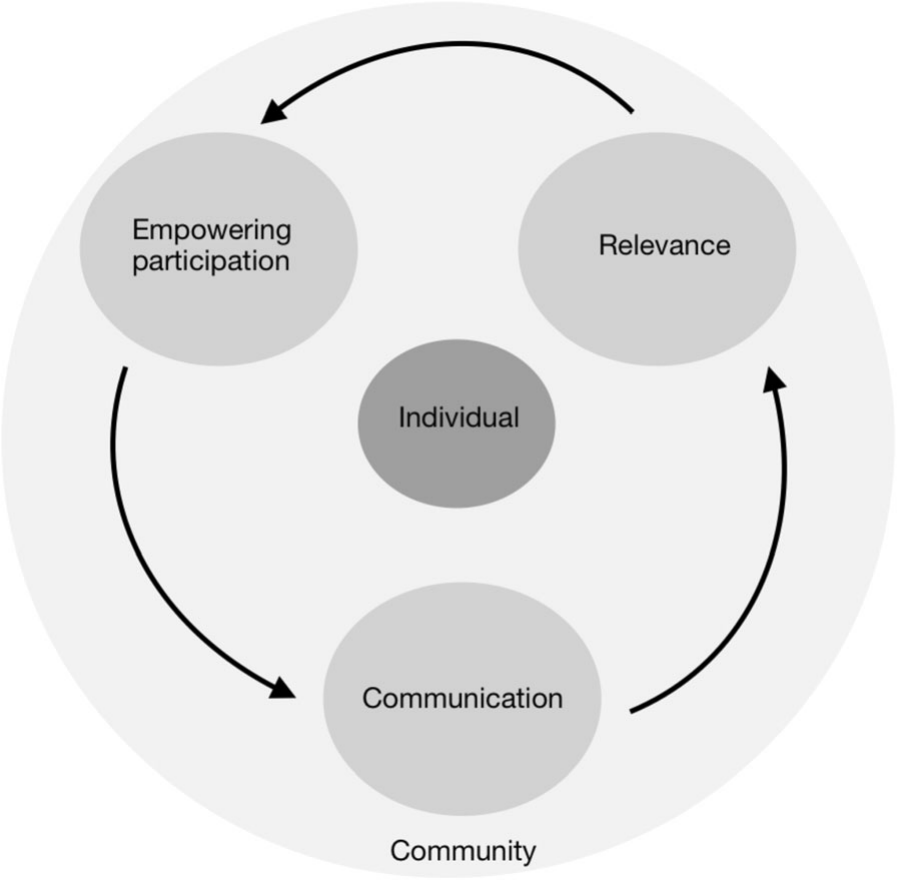
A framework for partnered population health research with rural and remote communities

Through our inductive thematic analysis, we identified three themes: *relevance*, *communication*, and *empowering participation* (Table 2). We have used these themes to develop a framework for community-partnered population health research in rural and remote communities that places the individual at the centre and frames identified principles within the community context (Fig. 1).

**Table 2:** Emergent themes identified from the perspectives of community members

Theme	Subthemes	Codes
Relevance	Community	• Benefit and value to community • Collaborative development • Community ownership and capacity building
	Individual	• Vested or personal interest • Opportunity to be involved and make impact for community
Communication	Use existing networks and create partnerships	• Identify local champions • Partner with existing organizations already doing the work in area of interest
	Clarify expectations	• Define project, role, and time commitment • Share progress and findings • Ensure information is accessible
	Identify preferred mechanism	• Understand local norms and context • Provide communication options for participation
Empowering participation	Being valued and appreciated	• Acknowledgement and recognition for contribution • Trust and relationship building
	Support	• Mentorship and training • Maintain environment of inclusiveness • Remove barriers to participation
	Application of research	• Evidence to support decision making • A tool to understand options and benefits for healthy living

### Relevance

Participants commonly stated that in order to engage as partners on research teams, the research project must be relevant to them as individuals and to their community. Individually, there must be an identified ‘hook’ or reason why the community member would engage in a specific project. This was reflected as the need for a vested interest based on personal meaning or related to their paid or volunteer work. Karen explains how it would be important to be able to justify the time required for participation on a research team:

*“…I think in order for people to commit to getting involved, they generally need something, some reason that they can and even sometimes just to justify the time…or just enough to make it worth their personal while” (Karen)*

Nearly all community members expressed how it is essential that research projects be relevant to and build capacity within their community. Participants stated that they would not be interested in partnering on projects if they did not see direct value or benefit for their communities. This highlights the need to incorporate community member perspectives from the initial stages of project development and to invest time to fully understand the priorities of communities so researchers are asking the right questions and measuring the right outcomes. As David and Janice describe, it is prudent that researchers consider who is in fact driving research questions:

*“…there’s a much more fundamental question and that is what is the research about? I don’t think that people are going to be interested in a research question that doesn’t apply to them so people would be engaged if they could see some point to the research and see that it applies in some way to their community and might lead to a benefit for their community… if it’s simply an academic kind of question that would be interesting to people in an academic setting I think it would be…very difficult to engage members of the community” (David)*

*“find out from your community what their biggest issue is with health…what would they like to see improving on… what are the obstacles…what would you like to see and how could we help you? (Janice)*

Participants emphasized that more than personal gain or compensation, community members need to anticipate a project will have direct benefit for their community. As described by participants, a collaborative or participatory research approach can lead to research outcomes that will be used by communities. Jane and Dave describe the need for research to have meaningful outcomes for communities to inspire change:

*“what’s it going to do for my community? Are the results being shared? Is it something that might inspire…or that gets something else going that we haven’t thought of” (Jane)*

*“I’m almost positive that people will not be interested in simply being approached about being engaged in research, unless they know what the research is about, and unless the research is relevant to their village and daily life” (David)*

### Communication

Participants expressed the need for regular communication as those who had been involved in research as either a participant or in facilitative role expressed that they often did not hear back about where things had gone or felt that the project had been shelved. Others expressed that because they were from a small community, their voices were washed out in comparison to larger communities.

*“we are a small community and a small voice…in the past what has happened is that we have been asked for our opinions on certain things, whether it’s governments coming in and saying, ‘hey we want to hear about what you’re doing and how we can help’ and then they say, ‘well gee, you only have a population of a thousand, so you don’t really have a lot of weight.’ So I think some people have had negative experiences where they do take the time, they do invest in projects, and then nothing comes out of it” (Steve)*

Participants noted aspects of communication related to both the recruitment and retention of community partners on research teams. For recruitment, it is essential to consider the community context and identify preferred communication mechanisms that would work best in that particular situation and environment. Several participants also noted the need for researchers to invest time to understand the context of each community and identify who is currently doing related work. Partnering with community leaders and using existing networks would help identify preferred mechanisms and assist researchers in understanding the local context.

For spreading of information, some participants recommend strategies such as word of mouth, sign-up sheets, email, and social media. Brenda suggested using the town council for assistance in identifying community champions:

*“We don’t have a newspaper, we have a very engaged radio, and council’s very engaged. So if you were looking at my community, you could do a presentation to council, this is the research we’re looking for and we’re looking for some key community members, and then they would come to you and say here’s some names that you should check because they know the community and they have a passion…then it’s kind of a prestigious thing, council recommended me to be a part of this research…they would be able to link you to the right community members” (Brenda)*

*“if you continue to reach out to the people that are actually on the ground doing the stuff...then you’re going to get that buy in…and you’ll get better results” (Brenda)*

Participants also frequently mentioned how it is essential to effectively communicate back to participants in a timely fashion, even just to confer that there are no updates, for example:

*“It’s just really about communication back … there might not be anything to communicate back to be honest, but it could be a simple hey just to let you know here’s an update” (Steve)*

*“It would be nice to have feedback… if it was helping in any way” (Judy)*

Relatedly, participants expressed the need to co-establish expectations for communication and clarity from the beginning regarding when and how information will be shared and, as Jane describes, to provide options for participating:

*“options for participating so if it’s inclement weather you can skype in that kind of thing … there are people of a certain age in a smaller community…winter sets in…they will drive when the weather is ok…but they are not going to venture out otherwise” (Jane)*

### Empowering participation

This theme indicates how important it is to invest time in building partnerships and relationships with community members. By forming an authentic partnership, providing adequate training, and identifying community members as subject matter experts, research teams can empower research engagement by patient and public partners. Participants reflected that researchers and health system decision makers often tend to arrive in their community, collect data or complete a superficial consultation on an issue, and leave – fracturing relationships. This issue of researchers ‘helicoptering’ into communities to collect data appears to be particularly relevant for engagement with rural and remote communities. Participants discussed how it is important that research comes from communities and work is done *with communities*, rather than *on communities* to support sustained health promotion interventions. Part of encouraging and supporting community members participation on research teams is understanding the application of research, which in turn can help facilitate the uptake of evidence into practice. This includes understanding how and why communities will use information, so that projects and outputs are developed to be most helpful and practical for communities after a single project is complete, as Brenda indicates:

*“…otherwise the buy in isn’t there and they’re like ‘oh here’s another person and they’ve got all these ideas and then they’re going to be gone in two years and everything’ll fall apart’ and, so you’ve got to create those relationships and say yeah…this is for you it’s not for me” (Brenda)*

This theme also includes the need to provide mentorship and training on the research process, the roles community members can play as research partners, and to clarify expectations. Community members in this study explained how it is essential to ensure everyone on the team understands their responsibilities to support and encourage sustained engagement:

*“maybe to have some sort of introduction to it…without necessarily biting off a huge amount” (Melissa)*

*“they might want a little bit of training about what’s going on and how the research works and stuff…cause if they didn’t understand the process they probably wouldn’t be as likely to stick with it” (Judy)*

Finally, the importance of valuing community members as subject matter experts was a recurring sentiment from participants in order to avoid the appearance or feeling of forced collaboration or tokenism, as Frank mentions:

*“you often want to battle that perception...you want to know that you’re there for your brain…you want to know that you’re there because you belong” (Frank)*

Theresa further commented on the need to appreciate team members:

*“I find once you get them there, appreciation keeps them there…appreciate them, thank them, recognize them” (Theresa)*

## Discussion

The goal of this project was to understand the needs and perspectives of community members from rural and remote areas to engage as partners on population health research teams. It is well known that for health promotion interventions to be successful, environmental, cultural, and social factors must be used to guide intervention development and implementation [28]. Public engagement throughout the research process is one way to ensure these contextual factors are fully incorporated into the research design and to support sustainability of interventions [29]. We present a co-produced framework that considers the three themes placed within a rural and remote community context. We also present our learnings from conducting this project following an integrated knowledge translation approach that includes researchers and knowledge users as full partners in study design, data collection, analysis, and interpretation.

Understanding how to partner with underrepresented voices and those who face increased barriers to health care is one strategy to address health inequities [13, 14]. This is only possible if the right people are given an opportunity to participate and shape research for their communities; the co-production of interventions can lead to more impactful health promotion strategies developed with communities, for communities [30]. For this process to occur, we must also understand mechanisms, principles, and guidelines to reach people who have typically been excluded. The results of this project indicate that, at the local level, understanding the applicability of research, measuring community-identified outcomes, and asking questions that are of value to community members will support research that is used by community members, helping to close the knowledge to action gap to ensure research is effectively translated into practice [7].

The results of this study echo other published reports of best practices for patient and public engaged research methods including respect, trust, providing training, and the need for regular bidirectional communication pathways [31]. Applying a deductive lens to our analysis based on the SPOR framework, we found similarities across the themes of communication and empowering participation. The identified need for community partners to have a personal relevance to the research topic is not indicated in the existing SPOR framework [1], and may reflect differences in public or community-centred and patient-oriented research. In patient-oriented research, personal relevance would be implied and related to the lived experience with a particular health issue that is under investigation. In population health research, although people would have experience and understanding of the contextual factors of their specific community, findings from this study indicate the need for personal relevance to the research topic (in this case, physical activity). It is not simply enough to ask for volunteers; there needs to be an effort on the part of the researcher to find a match between the individual and the opportunity. As mentioned by participants in the current study, identifying community champions related to a specific project or issue is best achieved through a focus on forming partnerships and understanding preferred local communication mechanisms [17]. Involving cultural or knowledge brokers is one approach to assist researchers to understand community context and identify people on the ground doing related work, this may be a particularly effective strategy when working with rural and remote communities that are a greater distance from researchers and where contextual factors are less well understood [32, 33].

A common sentiment expressed by participants were negative feelings related to researchers or health system decision makers coming in from larger urban centers, collecting data or conducting a tokenistic consultation on health system change, and leaving without providing adequate feedback. These feelings of discontent related to prior participation in research or the health system are commonly reported and may be related to a lack of perceived ownership. Community ownership and empowerment in research is best achieved with authentic partnerships, requires a transfer of power from researchers to communities, and the establishment of trust in the researcher [34]. Strategies to support community ownership of data, common in Indigenous and other participatory research approaches, include relationship building, equitable compensation, and sustained bidirectional communication with continual process evaluation [17, 32]. Specific characteristics on the part of the researcher such as willingness to spend time building relationships, humility, and determination to engage in community-centred research should also be considered [34]. These strategies would be well supported by successful implementation of the principles outlined in our findings: relevance, communication, and empowering participation.

The relatively high percentage of Indigenous people in northern BC and throughout Canada must also be carefully considered in the design of population health programming. Settler researchers must reflect on and acknowledge historical events, cultural barriers, intergenerational trauma, and institutional power imbalances that may contribute to a lack of trust in research partnerships. Although we did include participants who self-identified as Indigenous in this project, we did not complete an intentional analysis based on the perspectives of Indigenous people. There were comments by Indigenous and non-Indigenous participants alike about feelings of tokenism, “box-checking”, mistreatment and ethical violations with data, and using Indigenous culture and people to advance a researcher’s career. These findings are not unique [35]. We suggest understanding strategies to meaningfully and respectfully engage Indigenous research partners as an important area for future study; however, researchers would first need to be invited by a community and complete this work as part of an allied research paradigm that has direct value to communities [35–37].

Participants in this project had experience in research ranging from being research participants, to facilitating the recruitment and organization of local research sites, to participating in priority setting, and being knowledge-users on projects. We were inclusive in our recruitment so that anyone with experiences of research-like events (e.g. consultations) was invited to participate. The participants with more exposure to research were able to provide a more robust description of needs and strategies for engagement on research teams. Some participants with little to no exposure to research had more trouble imagining what they would need to participate or how we, as a research team, could best support that partnership. While this may indicate a general lack of understanding of the research process among community members, it also highlights the need for training and clarifying expectations in advance so potential community partners can make an informed decision about their participation.

### Limitations

A limitation of this project is that we did not include a community member partner on our research team. While this would have presented an opportunity for co-production, ultimately the inclusion of patient or public team members is the next step in our research, guided by the outcomes of this project as described in our framework. Although pragmatic, our recruitment method involved circulating an email through the existing physical activity networks of knowledge user team members. This strategy may have excluded some community members with divergent perspective from participating; however, it did enable a more robust and directed discussion with participants around a specific concept.

### Implications & Future Directions

Based on the outcomes and process of completing this partnered work, we propose four recommendations for researchers interested in conducting community-partnered population health research. Although some points raised by community members were specific to the context of northern BC, we recommend these universal principles for policy and practice:

1. Provide training for all team members. This would preferably be a workshop or engaged learning opportunity completed together with all team members, acknowledging that everybody has something to learn.
2. Understand the community context, preferred communication mechanisms, and identify community champions already engaged in the area of interest.
3. Ensure that every project asks questions and measures outcomes that are of interest to the community where research is taking place.
4. Acknowledge that community members are subject matter experts and ensure open, ongoing, two-way communication mechanisms.

For knowledge-users (including health system decision makers and health care providers) engaged on research teams, the takeaways from this project and recommendations for participating in partnered work include:

1. Establish and communicate a clear idea of what information you wish to collect as part of a research project, and how that information could be used (e.g., inform policy, practice, program and/or system change).
2. Be prepared with some potential research projects and questions to help jumpstart a partnership.
3. Learn and appreciate the unique contexts, assets and needs of each individual community, and establish connections with champions within each community. This would help to strengthen and sustain ongoing partnerships and communication over time, improve relevance and uptake of future engagements and interventions, and bring a unique contribution to the research team.

## Conclusions

The outcomes of this project add to a growing body of literature on patient and public engagement in research. Specifically, findings indicate that in order to meaningfully partner with members of the public in rural and remote communities, researchers must be attentive to the individual and community relevance of their work, establish ongoing bidirectional communication mechanisms that are appropriate to the community context, and value individuals as subject matter experts. Completing a similar project with participants after they have engaged on research teams may provide a different assessment of community members’ needs as patient and public research partners and offer more robust guidance for researchers. Creating a culture of open communication so that the perspectives of community members are respected and integrated into the research process is prudent and must be added into the timeline of individual research projects and programs of research.

## Supplementary information

**Additional file 1.** Plain language summary.

## Data Availability

The data generated and analysed during the current study are not publicly available due to confidentiality, but strictly anonymized versions of the data may be available from the corresponding author on reasonable request.

## Abbreviations

BC: British Columbia
CIHR: Canadian Institutes of Health Research
SPOR: Strategy for Patient Oriented Research
UNBC: University of Northern British Columbia
