# Supplementary material for: Exploring the perspectives of community members as research partners in rural and remote areas
Source: Res Involv Engagem. 2020 Jan 30;6:3. doi: 10.1186/s40900-020-0179-6 (PMC6990467; doi:10.1186/s40900-020-0179-6)
Supplement: Supplementary file 1 — Additional file 1. Plain language summary. [file 40900_2020_179_MOESM1_ESM.pdf]

How do we best partner with community members in physical activity research in northern BC?

Increasing participation in physical activity could help improve health in northern BC. When researchers partner with community members, it helps to make research more relevant and ensures we ask the right questions. In this study we were trying to understand how to better support and work with community members from across northern BC in physical activity research.

WHAT DID WE DO?

To find out how to engage community members, we interviewed 12 adults, all of whom have lived in northern BC for at least 1 year. We asked participants questions and discussed health research and physical activity. We used the interview results to identify main themes.

WHAT DID WE FIND?

We found 3 main factors that are important for research partnerships:

WHAT IS SPOR?

The Strategy for Patient-Oriented Research (SPOR) is a way of doing research where 'patients', or people who have personal experience with a particular health issue, work together with researchers to answer questions that are important to the patients and will lead to improvements in health or health care.

For more information check out:

https://bcsupportunit.ca/

https://www.msfhr.org/our-work/patient-oriented-research

1. The project must be **relevant** to the community and to the individuals taking part in it;
2. **Communication** between researchers and community members must be frequent, appropriate, and in a language that everyone understands;
3. Community members should be **empowered** as part of the research team through supports such as training, acknowledging and valuing their work, building strong relationships, and using the information from the project to support decision-making in the future.

WHAT NOW?

We will use this information as a guide to help us better partner with community members on our research team. Our overall research goal is to *Move the North*, increasing physical activity in northern BC, which can only be achieved if we work together.

For more information contact
Chelsea Pelletier (project lead):
Chelsea.Pelletier@unbc.ca or (250) 960-5283

UNBC UNIVERSITY OF NORTHERN BRITISH COLUMBIA

northern health
the northern way of caring

BC SUPPORT Unit
Advancing Patient-Oriented Research
NORTHERN CENTRE
